# Development and validation of prediction models for gestational diabetes treatment modality using supervised machine learning: a population-based cohort study

**DOI:** 10.1186/s12916-022-02499-7

**Published:** 2022-09-15

**Authors:** Lauren D. Liao, Assiamira Ferrara, Mara B. Greenberg, Amanda L. Ngo, Juanran Feng, Zhenhua Zhang, Patrick T. Bradshaw, Alan E. Hubbard, Yeyi Zhu

**Affiliations:** 1 Division of Biostatistics, School of Public Health, University of California, Berkeley, CA USA; 2 Division of Research, Kaiser Permanente Northern California, Oakland, CA USA; 3 Department of Obstetrics and Gynecology, Kaiser Permanente Northern California, Oakland, CA USA; 4 Regional Perinatal Service Center, Kaiser Permanente Northern California, Santa Clara, CA USA; 5 Department of Civil and Environmental Engineering, Stanford University, Palo Alto, CA USA; 6 Division of Epidemiology, School of Public Health, University of California, Berkeley, CA USA; 7 Department of Epidemiology and Biostatistics, University of California, San Francisco, CA USA

**Keywords:** Gestational diabetes, Machine learning, Pharmacologic treatment, Prediction, Pregnancy, Risk stratification, Treatment modality

## Abstract

**Background:**

Gestational diabetes (GDM) is prevalent and benefits from timely and effective treatment, given the short window to impact glycemic control. Clinicians face major barriers to choosing effectively among treatment modalities [medical nutrition therapy (MNT) with or without pharmacologic treatment (antidiabetic oral agents and/or insulin)]. We investigated whether clinical data at varied stages of pregnancy can predict GDM treatment modality.

**Methods:**

Among a population-based cohort of 30,474 pregnancies with GDM delivered at Kaiser Permanente Northern California in 2007–2017, we selected those in 2007–2016 as the discovery set and 2017 as the temporal/future validation set. Potential predictors were extracted from electronic health records at different timepoints (levels 1–4): (1) 1-year preconception to the last menstrual period, (2) the last menstrual period to GDM diagnosis, (3) at GDM diagnosis, and (4) 1 week after GDM diagnosis. We compared transparent and ensemble machine learning prediction methods, including least absolute shrinkage and selection operator (LASSO) regression and super learner, containing classification and regression tree, LASSO regression, random forest, and extreme gradient boosting algorithms, to predict risks for pharmacologic treatment beyond MNT.

**Results:**

The super learner using levels 1–4 predictors had higher predictability [tenfold cross-validated C-statistic in discovery/validation set: 0.934 (95% CI: 0.931–0.936)/0.815 (0.800–0.829)], compared to levels 1, 1–2, and 1–3 (discovery/validation set C-statistic: 0.683–0.869/0.634–0.754). A simpler, more interpretable model, including timing of GDM diagnosis, diagnostic fasting glucose value, and the status and frequency of glycemic control at fasting during one-week post diagnosis, was developed using tenfold cross-validated logistic regression based on super learner-selected predictors. This model compared to the super learner had only a modest reduction in predictability [discovery/validation set C-statistic: 0.825 (0.820–0.830)/0.798 (95% CI: 0.783–0.813)].

**Conclusions:**

Clinical data demonstrated reasonably high predictability for GDM treatment modality at the time of GDM diagnosis and high predictability at 1-week post GDM diagnosis. These population-based, clinically oriented models may support algorithm-based risk-stratification for treatment modality, inform timely treatment, and catalyze more effective management of GDM.

**Supplementary Information:**

The online version contains supplementary material available at 10.1186/s12916-022-02499-7.

## Background

As the most common metabolic complication during pregnancy, gestational diabetes mellitus (GDM) has increased in prevalence by 33–90% over the past decades and is currently affecting 6–12% pregnancies across the globe [1, 2]. GDM predisposes individuals and their children to a multitude of perinatal and long-term sequelae of cardiovascular and neurodevelopmental complications, forming a growing, urgent public health concern [3]. Barriers to optimizing care of the large group of affected patients include timing of conventional screening and diagnosis towards late pregnancy and multiple lines of therapy, leaving little time for effective treatment.

According to American College of Obstetrics and Gynecology (ACOG) and American Diabetes Association (ADA) guidelines [4, 5], individuals with GDM should universally receive medical nutrition therapy (MNT) as the first-line therapy. If optimal glycemic control is not achieved, more resource-intensive pharmacologic treatment is added to MNT. This process may take several weeks during which individuals and their fetus continue to be exposed to hyperglycemia [6]. Therefore, efficient care relies on timely risk stratification for GDM treatment modality, which would enable early triage to be incorporated into risk-based models of care and allow early initiation of efficacious treatment [7]. Indeed, there has been increasing interest in developing risk prediction tools for GDM treatment modality.

Although insulin is the standard pharmacologic treatment for GDM approved by the US Food and Drug Administration, use of antidiabetic oral agents, such as glyburide and metformin, has increased dramatically over the past decades [8]. Given notable advantages of the ease of use, lower cost, and acceptance among patients [9, 10], the prevalence of glyburide use for GDM treatment increased from 7.4% in 2000 to 64.5% in 2011 in the USA; antidiabetic oral agents have replaced insulin as the more common pharmacotherapy for GDM over the past decades [8]. Nonetheless, risk prediction models to discriminate MNT versus additional pharmacologic treatment including both antidiabetic oral agents and/or insulin are lacking.

To address these critical clinical data gaps about risk stratification for treatment modality among pregnant individuals with GDM and promptly starting the needed treatment, we aimed to develop predictive models using supervised machine learning algorithms based on clinically available factors at varied time points spanning from 1 year prior to pregnancy to 1-week post GDM diagnosis to predict individuals in need of intensive pharmacologic treatment (i.e., antidiabetic oral agents and/or insulin) beyond MNT.

## Methods

### Study population and design

The study population was drawn from the membership of Kaiser Permanente Northern California (KPNC), an integrated health care delivery system serving 4.5 million members. The KPNC membership accounts for approximately 30% of the underlying population and is socio-demographically representative of the population residing in the geographic areas served [11, 12]. The integrated information system permits quantifying predictors and outcomes across the continuum of pregnancy. Individuals with GDM are identified by searching the KPNC Pregnancy Glucose Tolerance and GDM Registry, which is an active surveillance registry that downloads laboratory data to determine screening and diagnosis for GDM, where preexisting type 1 or 2 diabetes is automatically excluded. Specifically, pregnant individuals at KPNC receive universal screening (98%) for GDM with the 50-g, 1-h glucose challenge test (GCT) at 24–28 weeks’ gestation [1]. If the screening test is abnormal, a diagnostic 100-g, 3-h oral glucose tolerance test (OGTT) is performed after an 8–12-h fast. GDM is ascertained by meeting any of the following criteria: (1) ≥ 2 OGTT plasma glucose values meeting or exceeding the Carpenter-Coustan thresholds: 1-h 180 mg/dL, 2-h 155 mg/dL, and 3-h 140 mg/dL; or (2) 1-h GCT ≥ 180 mg/dL and a fasting glucose ≥ 95 mg/dL performed alone or during the OGTT [13, 14]. Plasma glucose measurements were performed using the hexokinase method at the KPNC regional laboratory, which participated in the College of American Pathologists’ accreditation and monitoring program [15]. This data-only project was approved by the KPNC Institutional Review Board, which waived the requirement for informed consent from participants.

**Fig. 1:**
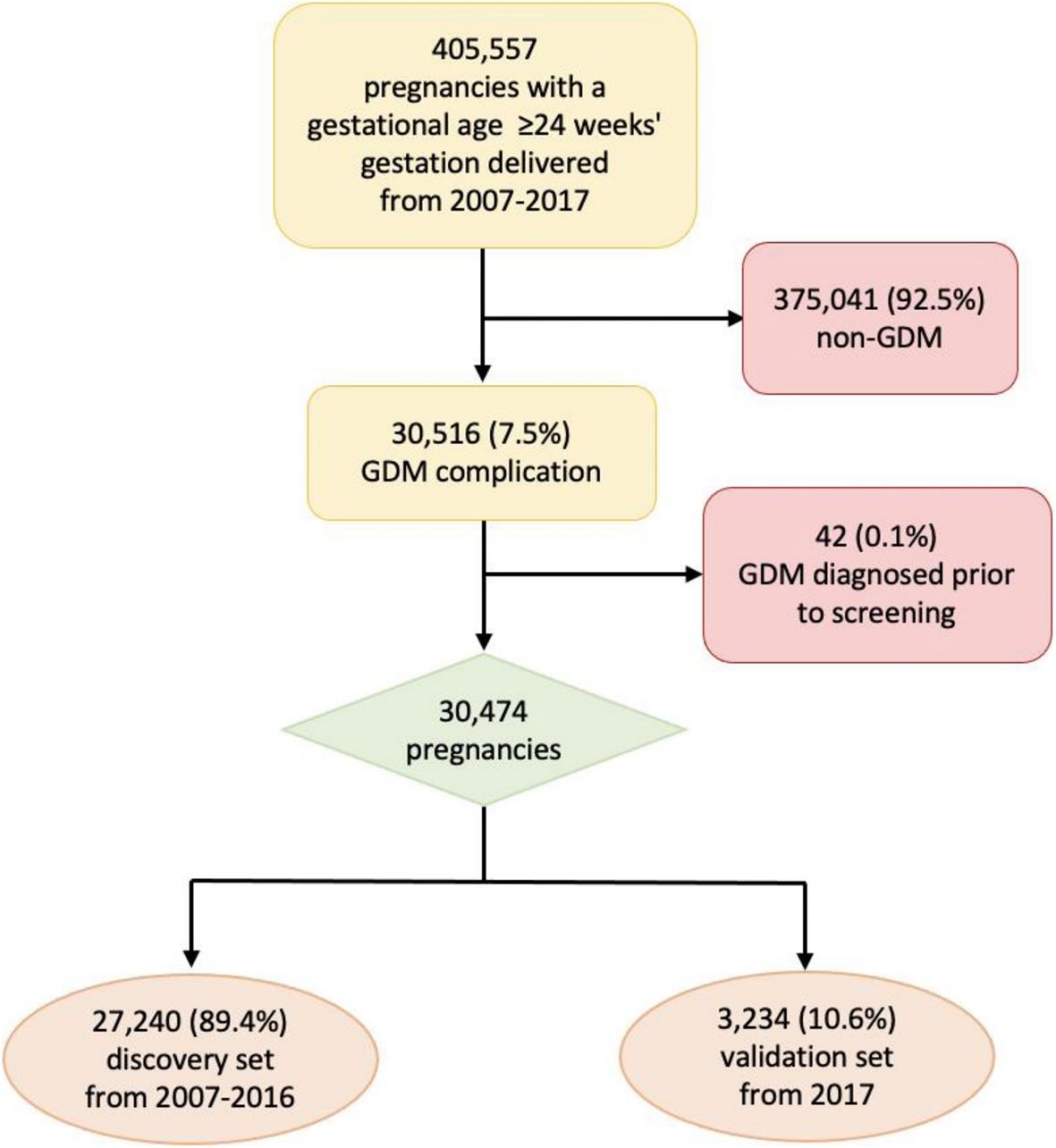
Flowchart for developing pregnancies cohort with gestational diabetes 2007–2017. GDM,: gestational diabetes mellitus

Among 405,557 pregnancies with a gestational age at delivery < 24 weeks’ gestation delivered at 21 KPNC hospitals from January 1, 2007, to December 31, 2017, we excluded 375,041 (92.5%) individuals without GDM. Among 30,516 GDM pregnancies, we further excluded individuals with GDM diagnosed before the universal GDM screening (*n* = 42), deriving an analytical sample of 30,474 GDM-complicated pregnancies. We further derived a discovery set containing 27,240 GDM-complicated pregnancies from 2007 to 2016 and a temporal/future validation set of 3234 GDM-complicated pregnancies in 2017 (Fig. 1).

### Outcome ascertainment

Individuals diagnosed with GDM received universal referral to the KPNC Regional Perinatal Service Center for the supplemental care program beyond their standard of prenatal care. MNT was the first-line therapy. If glycemic control targets were not achieved with MNT alone, pharmacologic treatment was initiated. Based on counseling regarding risks and benefits of antidiabetic oral agents versus insulin, pharmacologic treatment was chosen via a patient-physician shared decision-making model: (1) with antidiabetic oral agents such as glyburide and metformin being added to MNT and if optimal glycemic control continued to fail, oral medication was escalated to insulin therapy, and (2) or with insulin therapy initiated directly beyond MNT (an additional table shows this in more detail [see Additional file 1]). We searched the pharmacy information management database for prescriptions for oral agents (glyburide 97.9%, metformin or other) and insulin after GDM diagnosis. Treatment modality was grouped as MNT only and pharmacologic treatment (oral agents and/or insulin) beyond MNT. Notably, despite an overall large sample size, we grouped oral agents (32.6% of the entire population) and insulin (6.2%) into pharmacologic treatment due to insufficient power to predict insulin separately as an outcome.

### Candidate predictors

Based on risk factors associated with GDM treatment modality and input from clinicians, we selected 176 (64 continuous and 112 categorical) sociodemographic, behavioral, and clinical candidate predictors obtained from electronic health records for model development. Candidate predictors were divided into four levels based on availability at varied stages of pregnancy (an additional table shows this in more detail [see Additional file 2]): Level 1 predictors (*n* = 68) were available at the initiation of pregnancy and dated back to 1 year prior to the index pregnancy; level 2 predictors (*n* = 26) were measured from the last menstrual period to before GDM diagnosis; level 3 predictors (*n* = 12) were available at the time of GDM diagnosis; and level 4 (*n* = 70) included self-monitoring of blood glucose (SMBG) levels, as the primary measure of glycemic control during pregnancy as recommended by the American Diabetes Association [5], measured the first week after the GDM diagnosis. All predictors, levels 1–4, were measured prior to the outcome of interest (i.e., final line of GDM treatment). Pregnant individuals with GDM in our study population had on average, 11.8 weeks (standard deviation: 6.6 weeks), of SMBG measurements between GDM diagnosis and delivery. We included data 1 week after GDM diagnosis to allow earlier prediction since it takes on average 5.6 weeks between GDM diagnosis and the optimal treatment is offered. Of note, individuals with GDM were universally offered enrollment to a supplemental GDM care program managed by nurses and dietitians via telemedicine from the KPNC Regional Perinatal Service Center [16]. All individuals with GDM were instructed to self-monitor and record glucose measurements four times per day: fasting before breakfast and 1 h after the start of each meal. Measurements of SMBG were then reported to the nurses or registered dieticians during weekly telephone counseling calls from enrollment until delivery and data were recorded in the Patient Reported Capillary Glucose Clinical Database.

### Statistical analysis

#### Preprocessing

We imputed missing values with the random forest algorithm since the algorithm does not require parametric model assumptions, which reduce the efficiency of the predictor (an additional table shows this in more detail [see Additional file 2]). We evaluated the estimation of true imputation error using normalized root mean squared error and proportion of falsely classified entries for continuous and categorical variables, respectively. Both values were close to 0, indicating good performance in imputation (an additional table shows this in more detail [see Additional file 3]). After preprocessing, we employed *t*-test and Pearson’s chi-squared test to compare participant characteristics between the discovery and temporal/future validation sets. We conducted the Mann–Kendall test to examine secular trends for GDM treatment modalities across calendar years. The discovery set (2007–2016) was stratified by the calendar year and treatment modality for tenfold cross validation. The temporal/future validation set (2017) was stratified by treatment modality for cross-validated prediction performance computation.

#### Variable selection and full model development and comparison

We performed prediction through classification and regression tree (CART), least absolute shrinkage and selection operator (LASSO) regression, and super learner (SL) predicting with levels 1, 1–2, 1–3, and 1–4 predictors, respectively. CART and LASSO regression were chosen as simple prediction methods compared to SL. The SL defines a set of candidate machine learning algorithms, namely, the library, and combines prediction results through meta-learning via cross-validation [17]. SL has the asymptotic property that it is at least as good (in risk, defined by the negative log-likelihood) as the best fitting algorithm in the library [17]. Although the variables included in the final ensemble SL cannot be easily interpreted for their individual contributions, SL can be used for optimal prediction performance and to benchmark simpler and less adaptive approaches [17].

We tuned the prediction methods as follows. In CART, the Gini index measured the heterogeneity composition of the subset with respect to the outcome, and maximum depth (6) was defined as the stopping criterion. Accounting for potential errors from the risk curve estimation, the regularization parameter in LASSO regression was selected from the cross-validated error within one standard error of its minimum value [18]. For the SL, we considered a simple and a complex library for comparison. The simple library included the response-mean, LASSO regression, and CART; the complex library expanded by additionally including random forest and extreme gradient boosting (XGBoost). Multiple XGBoosts were considered, where their tuning parameters were set to 10, 20, 50 trees, 1 to 6 maximum depths, and 0.001, 0.01, and 0.1 shrinkage for regularization.

For models using predictors at each level, prediction results were evaluated using tenfold cross-validated receiver operating characteristic curves and area under the receiver operating characteristic curve (AUC) statistics in the discovery and temporal/future validation sets. We used Delong’s test to compare AUCs between different prediction algorithms at the same predictor level and within the same prediction algorithm across levels, respectively [19]. We used permutation-based variable importance to calculate the AUCs with 5 simulations and obtained the top 10 important features. Permuting one variable at a time, the method calculated the AUC difference before and after permutation to assign an importance measure [20]. The model with the highest AUC in the validation set was selected as the final full model.

#### Development of simpler models

To improve interpretability and potential clinical uptake, we used tenfold cross-validated logistic regression to develop simpler models in the discovery set based on a minimal set of the most important features at each level, as opposed to the full set of features used in the complex SL. We additionally selected interaction term(s) considering all cross-products through stepwise forward and backward selection by the Akaike information criterion. We evaluated the predictive performance (i.e., simplicity and cross-validated AUCs) of these simpler models on the validation set. Further, calibration was examined by evaluating the quality of an uncalibrated model via the integrated calibration index, which captured the distribution of predicted probabilities, coupled with a calibration plot. Calibration method (i.e., isotonic regression) was implemented for recalibration in the event of observed over- or under-prediction.

## Results

Compared to 27,240 individuals with GDM in the discovery set, those in the temporal/future validation set (*n* = 3234) were slightly older but did not meaningfully differ by other characteristics, despite statistical significance due to the large sample size (Table 1). There was an overall increasing trend of antidiabetic oral agents use from 2007 to 2017 (*P*-for-trend = 0.0003) but not for MNT only or insulin therapy (an additional table shows this in more detail [see Additional file 4]). Among 11,817 (38.8%) individuals who received pharmacologic treatment beyond MNT for GDM, the mean time from the first-line MNT to the final antidiabetic oral agents and/or insulin therapy initiation was 5.6 weeks (standard deviation: 4.3 weeks), highlighting the significant time lapse between the first-line MNT to the last-line pharmacotherapy.

**Table 1:** Characteristics of individuals with gestational diabetes at Kaiser Permanente Northern California, 2007–2017

	**All (2007–2017)**	**Discovery set (2007–2016)**	**Temporal validation set (2017)**	***P* value**^**1**^
	***n* = 30,474**	***n* = 27,240**	***n* = 3234**	
Age at childbirth, mean (SD), y				< 0.001
15–24	1624 (5.3)	1497 (5.5)	127 (3.9)	
25–29	6057 (19.9)	5522 (20.3)	535 (16.5)	
30–34	11,295 (37.1)	10,018 (36.8)	1277 (39.5)	
≥ 35	11,498 (37.7)	10,203 (37.5)	1295 (40.0)	
Race/Ethnicity, *n* (%)				0.028
White	6866 (22.5)	6174 (22.7)	692 (21.4)	
Hispanic	8506 (27.9)	7655 (28.1)	851 (26.3)	
African American	1319 (4.3)	1174 (4.3)	145 (4.5)	
Asian/Pacific Islander	12,377 (40.6)	10,990 (40.3)	1387 (42.9)	
Other	1406 (4.6)	1247 (4.6)	159 (4.9)	
Pre**-**pregnancy body mass index, kg/m^2^, *n* (%)				< 0.001
Underweight	399 (1.3)	344 (1.3)	55 (1.7)	
Normal	6850 (22.5)	6147 (22.6)	703 (21.7)	
Overweight	10,095 (33.1)	9106 (33.4)	989 (30.6)	
Obese	13,130 (43.1)	11,643 (42.7)	1487 (46.0)	
Median household income, annual, *n* (%)				< 0.001
< $25,000	813 (2.7)	562 (2.1)	251 (7.8)	
$25,000–39,999	2816 (9.2)	2495 (9.2)	321 (9.9)	
$40,000–59,999	7169 (23.5)	6463 (23.7)	706 (21.8)	
$60,000–79,999	7796 (25.6)	7010 (25.7)	786 (24.3)	
≥ $80,000	11,880 (39.0)	10,710 (39.3)	1170 (36.2)	
Nulliparity, n (%)	12,419 (40.8)	11,117 (40.8)	1302 (40.3)	0.559
Gestational age at delivery, mean (SD), weeks	38.3 (1.9)	38.3 (1.9)	38.2 (1.9)	0.05

^1^Obtained by Student’s *t* test for continuous variables or Pearson’s chi-squared test for categorical variables

### Machine learning prediction methods comparison at different timings

Across the four timings, the tenfold cross-validated AUCs in the discovery and validation sets were overall lowest with level 1 predictors from 1-year preconception to LMP, regardless of prediction methods (Table 2). The models adding level 2 predictors performed slightly better than those at level 1 (an additional table shows this in more detail [see Additional file 5]). For models at levels 1–2, the complex SL (an additional table shows this in more detail [see Additional file 6]) outperformed the simple SL (an additional table shows this in more detail [see Additional file 7]), followed by LASSO regression and CART (tenfold cross-validated AUCs in the discovery set: 0.761, 0.688, 0.685, 0.618, respectively; Table 2). Adding level 3 predictors at GDM diagnosis increased the AUCs by approximately 0.10 across all models in both the discovery and validation sets; the highest AUC (95% CI) was observed for the complex SL [tenfold cross-validated AUC in the discovery set: 0.869 (0.865–0.873); validation set: 0.754 (0.739–0.772)]. The addition of level 4 predictors, 1 week after GDM diagnosis, further increased AUCs by approximately 0.05–0.07 across models, with the highest performance by the complex SL [discovery: 0.934 (0.931–0.936); validation: 0.815 (0.800–0.829)].

**Table 2:** Area under the receiver operating characteristic curve prediction results predictors at varied stages of pregnancy

Predictor levels^a^	Dataset	AUC (95% CI)			
		**CART**	**LASSO regression**	**Simple super learner**^b^	**Complex super learner**^**c**^
1	Discovery set	0.613 (0.603–0.622)	0.670 (0.663–0.676)	0.673 (0.667–0.679)	0.683 (0.676–0.689)
	Validation set	0.592 (0.567–0.616)	0.634 (0.615–0.653)	0.635 (0.615–0.654)	0.634 (0.615–0.653)
1, 2	Discovery set	0.618 (0.609–0.628)	0.685 (0.678–0.691)	0.688 (0.682–0.695)	0.761 (0.756–0.767)
	Validation set	0.588 (0.563–0.613)	0.647 (0.628–0.666)	0.645 (0.626–0.664)	0.648 (0.630–0.667)
1, 2, 3	Discovery set	0.740 (0.732–0.748)	0.785 (0.780–0.791)	0.790 (0.785–0.796)	0.869 (0.865–0.873)
	Validation set	0.703 (0.682–0.724)	0.750 (0.733–0.767)	0.749 (0.733–0.766)	0.754 (0.739–0.772)
1, 2, 3, 4	Discovery set	0.785 (0.777–0.792)	0.849 (0.845–0.854)	0.852 (0.848–0.857)	0.934 (0.931–0.936)
	Validation set	0.745 (0.722–0.767)	0.809 (0.794–0.823)	0.808 (0.794–0.823)	0.815 (0.800–0.829)

*AUC*, area under the receiver operating characteristic curve; *CART*, classification and regression tree; *LASSO*, least absolute shrinkage and selection operator

^a^Level 1: 1-year preconception to last menstrual period; level 2: last menstrual period to before diagnosis of gestational diabetes; level 3: at the time of diagnosis of gestational diabetes; level 4: 1 week after diagnosis of gestational diabetes

^b^Candidate algorithms in simple super learner included response-mean, LASSO regression, and CART

^c^Candidate algorithms in complex super learner included response-mean, LASSO regression, CART, random forest, and extreme gradient boosting

### Most influential features or predictors

**Fig. 2:**
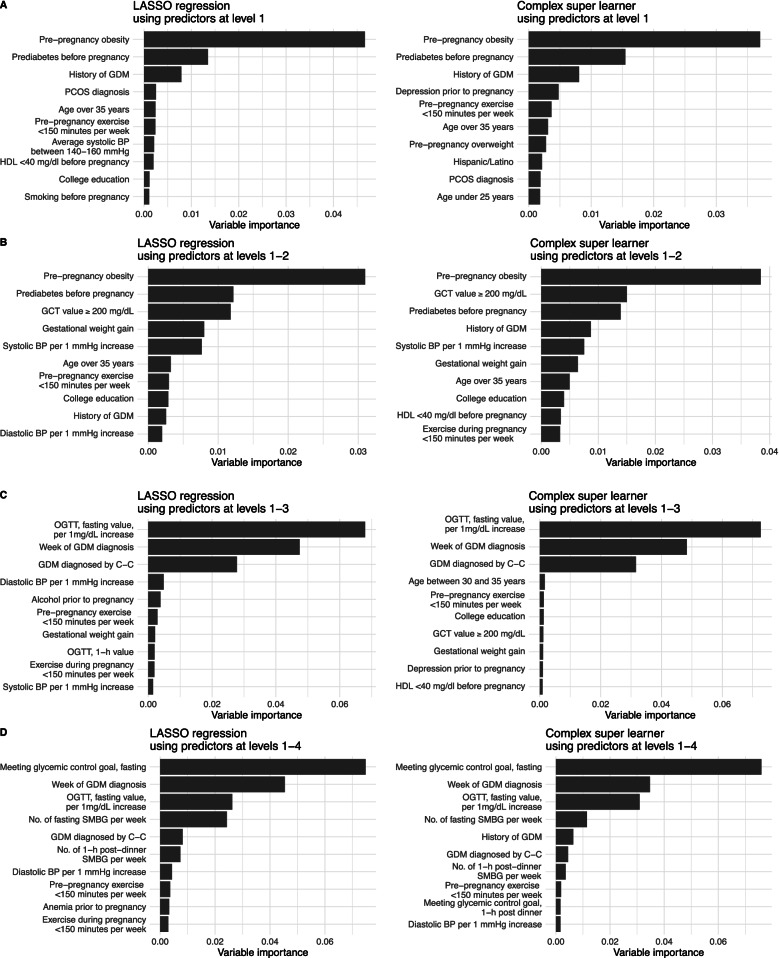
Variable importance for predictors at level(s) **A** 1, **B** 1–2, **C** 1–3, and **D** 1–4. BP, blood pressure; C–C, Carpenter-Coustan’s criteria; GCT, glucose challenge test; GDM, gestational diabetes mellitus; HDL, high-density lipoprotein; LASSO, least absolute shrinkage and selection operator; OGTT, oral glucose tolerance test; PCOS, polycystic ovary syndrome; SMBG, self-monitored blood glucose. Level 1: 1-year preconception to last menstrual period; level 2: last menstrual period to before diagnosis of gestational diabetes; level 3: at the time of diagnosis of gestational diabetes; level 4: 1 week after diagnosis of gestational diabetes

For level 1 predictors at the initiation of pregnancy (Fig. 2A; an additional figure shows this in more detail [see Additional file 8]), the top three contributors to the prediction based on variable importance were the same across prediction methods: pre-pregnancy obesity, prediabetes before pregnancy, and history of GDM. For predictors at levels 1–2 (Fig. 2B; an additional figure shows this in more detail [see Additional file 8]), the top four features in CART and simple and complex SL included the top three at level 1 with the addition of glucose levels at GCT for GDM screening (≥ 200 mg/dL).

At the timing of GDM diagnosis with the addition of level 3 predictors, OGTT fasting glucose value (per 1 mg/dL increase), gestational week at GDM diagnosis (continuous), and GDM diagnosis by Carpenter-Coustan criteria (versus by fasting hyperglycemia) were consistently the top three features across prediction methods (Fig. 2C; an additional figure shows this in more detail [see Additional file 8]). Adding level 4 predictors (Fig. 2D; an additional figure shows this in more detail [see Additional file 8]), the top four consistently selected by LASSO regression and the simple and complex SL were self-monitored glycemic control status at fasting, gestational week at GDM diagnosis (continuous), OGTT fasting glucose value (per 1 mg/dL increase), and number of fasting self-monitored blood glucose measurements. In the complex SL, the difference between importance measures of the fourth and fifth (i.e., history of GDM) top features was substantial (approximately by 40%); thus, the fifth-ranked and below predictors were not included as top features for the development of simpler models (see below).

### Development and calibration of simpler models

We constructed a series of simpler logistic regression models with a minimum set, as opposed to the full set, of predictors at each timing to balance the model predicative performance and feasibility and easiness of clinical implementation and uptake. The main predictors of the simpler models were selected via the complex SL, and we further included interaction terms selected from the optimal stepwise logistic regression (an additional table shows this in more detail [see Additional file 9]). At pregnancy initiation, the simpler model using level 1 predictors identified history of GDM, pre-pregnancy obesity, and prediabetes before pregnancy [tenfold cross-validated AUC in the discovery set: 0.632, 95% CI (0.623–0.640); validation set AUC: 0.609 (0.587–0.632); Table 3]. The simpler model at levels 1–2 included history of GDM, pre-pregnancy obesity, glucose levels at GCT for GDM screening (≥ 200 mg/dL), and prediabetes before pregnancy, in addition to three pairwise interactions between the first three predictors, with similar AUC (95% CI) to that at level 1 [discovery set AUC: 0.648 (0.640–0.656); validation set AUC: 0.621 (0.599–0.643)]. At GDM diagnosis, the model using levels 1–3 predictors included OGTT fasting glucose value, gestational week at GDM diagnosis, and GDM diagnosis by Carpenter-Coustan criteria [discovery set AUC: 0.770 (0.764–0.775); validation set AUC: 0.746 (0.730–0.763)]. One week after GDM diagnosis, the simpler model included gestational week at GDM diagnosis, OGTT fasting glucose value, self-monitored glycemic control status at fasting, number of fasting self-monitored blood glucose measurements, and an interaction term between last two variables [discovery set AUC: 0.825 (0.820–0.830); validation set AUC: 0.798 (0.783–0.813)]. The simpler logistic regression models used for prediction is shown in Table 4.

**Table 3:** Prediction results using final simplified logistic regression models with predictors at varied stages of pregnancy

	**Cross-validated AUC (95% CI)**		**Integrated calibration index**	**Calibrated AUC (95% CI)**
	**Discovery set**	**Validation set**		
Level 1^a^	0.632 (0.623–0.640)	0.609 (0.587–0.632)	0.073	0.609 (0.587–0.632)
Levels 1–2^b^	0.648 (0.640–0.656)	0.621 (0.599–0.643)	0.075	0.621 (0.599–0.643)
Levels 1–3^c^	0.770 (0.764–0.775)	0.746 (0.730–0.763)	0.072	0.752 (0.734–0.77)
Levels 1–4^d^	0.825 (0.820–0.830)	0.798 (0.783–0.813)	0.038	0.802 (0.786–0.818)

*AUC*, area under the receiver operating characteristic curve; *CI*, confidence interval; *GDM*, gestational diabetes

^a^Predictors included history of GDM, pre-pregnancy obesity, and prediabetes before pregnancy

^b^Predictors included history of GDM, pre-pregnancy obesity, glucose levels at 50-g, 1-h glucose challenge test for GDM screening (≥ 200 mg/dL), and prediabetes before pregnancy, in addition to three pairwise interactions between the first three predictors

^c^Predictors included fasting glucose value at 100-g, 3-h oral glucose tolerance test, gestational week at GDM diagnosis (continuous), and GDM diagnosis by Carpenter-Coustan criteria (versus by fasting hyperglycemia)

^d^Predictors included gestational week at GDM diagnosis (continuous), fasting glucose value at 100-g, 3-h oral glucose tolerance test, self-monitored glycemic control status at fasting, number of fasting self-monitored blood glucose measurements, and an interaction term between last two variables

**Table 4:** Final models developed by simplified logistic regression

Level 1	− 0.856 to 0.005 * history of GDM + 0.741 * BMI obese + 0.800 * prediabetes before pregnancy
Levels 1–2	− 1.001 + 0.572 * history of GDM + 0.579 * pre-pregnancy obesity + 0.774 * prediabetes before pregnancy + 0.733 * screening value^a^ − 0.323 * history of GDM * pre-pregnancy obesity − 0.577 * history of GDM * screening value^a^ + 0.480 * pre-pregnancy obesity * screening value^a^
Levels 1–3	− 4.468 + 0.074 * oral glucose tolerance test^b^ − 0.063 * week of gestational age^c^ − 1.435 diagnosis by C–C criteria^d^
Levels 1–4	− 2.645 to 0.810 * meeting glycemic control goal^e^ + 0.167 * number of SMBG tests taken − 0.076 * week of gestational age^c^ + 0.044 * oral glucose tolerance test^b^ − 0.234 * meeting glycemic control goal^e^ * number of SMBG tests taken

*BMI*, body mass index; *GDM*, gestational diabetes mellitus; *SMBG*, self-monitored blood glucose

The outcome is in log odds form, and coefficients have been rounded to the third decimal point

^a^Glucose levels at 50-g, 1-h glucose challenge test for GDM screening (≥ 200 mg/dL)

^b^Fasting glucose value at 100-g, 3-h oral glucose tolerance test

^c^Gestational week at GDM diagnosis (continuous)

^d^GDM diagnosis by Carpenter-Coustan criteria (versus by fasting hyperglycemia)

^e^SMBG control status for the fasting test measured during first week after GDM diagnosis

**Fig. 3:**
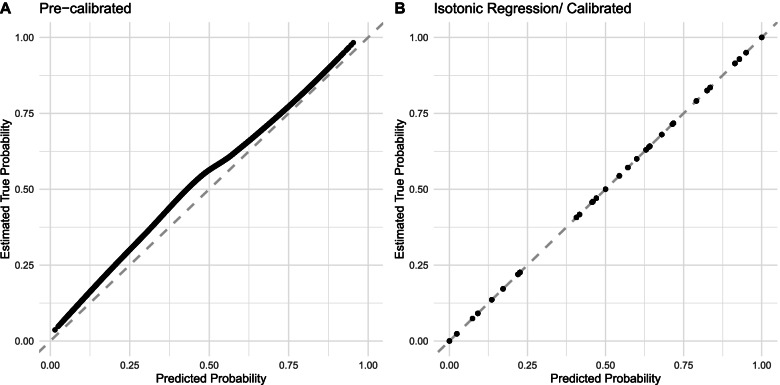
Pre- and post-calibration plots using logistic regression with level 1–4 predictors on the validation set. The simpler logistic regression model included gestational week at diagnosis of gestational diabetes, the diagnostic fasting glucose value, the status and frequency of self-monitored glycemic control at fasting during 1-week post diagnosis, and an interaction term of the last two variables. The dashed line indicates a perfectly calibrated model

**Fig. 4:**
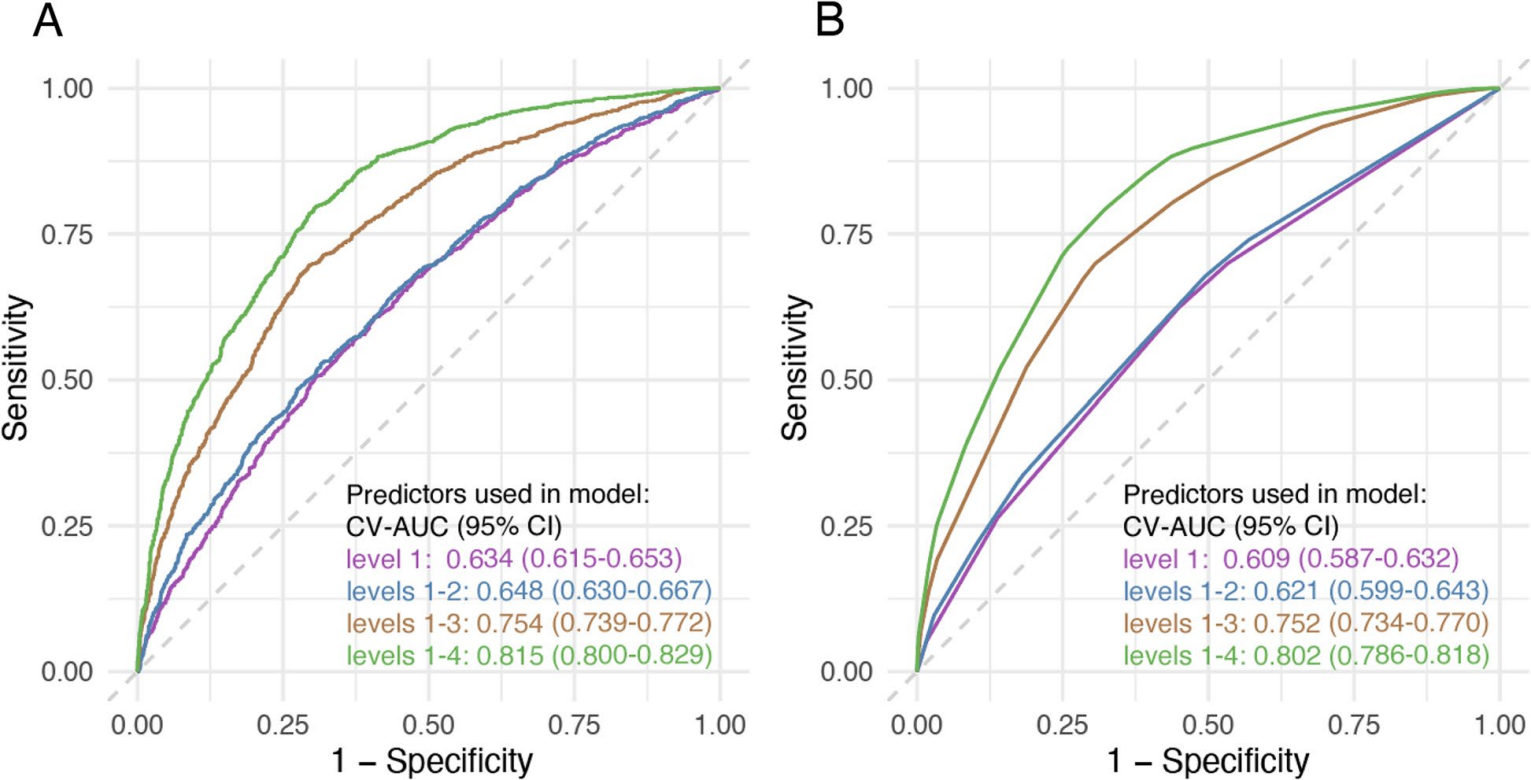
Prediction results from **A** complex super learner and **B** logistic regression at varied pregnancy stages. (1) Complex super learner algorithm included response-mean, LASSO regression, CART, random forest, and extreme gradient boosting. The simpler logistic regression models were developed based on predictors selected in the complex super learner algorithms at each level, aiming to include a minimum set of predictors for easier interpretability and higher clinical uptake. (2) Level 1: 1-year preconception to last menstrual period; level 2: last menstrual period to before diagnosis of gestational diabetes; level 3: at the time of diagnosis of gestational diabetes; level 4: 1 week after diagnosis of gestational diabetes. (3) The corresponding difference in AUC by Delong’s test between the complex super learner and simpler logistic regression models using level 1, levels 1–2, levels 1–3, and levels 1–4 are 0.073, 0.049, 0.831, and 0.264 respectively. AUC, area under the receiver operating characteristic curve; LASSO: least absolute shrinkage and selection operator

We further evaluated calibration performance of simpler models at varied levels in the temporal/future validation set, which indicated a slight difference between the predicted risk and the estimated true probability (integrated calibration index: 0.073, 0.074, 0.072, and 0.038 at each level, respectively; Table 3). In the pre-calibrated plot of the simpler model using level 1–4 predictors (Fig. 3A), the predicted probability was slightly underestimating the estimated true probability. After the isotonic regression calibration (Fig. 3B), the cross-validated AUC in the validation set increased slightly (0.802, 95% CI: 0.786–0.818). The performance of calibrated simpler models was comparable to that of the complex SL on the validation set (Fig. 4).

## Discussion

In this population-based cohort study of 30,474 multi-racial/ethnic pregnant individuals with GDM, we predicted GDM treatment modality (pharmacologic treatment vs. MNT only) with diverse supervised machine learning algorithms. Using predictors from 1-year before the index pregnancy through 1-week post GDM diagnosis, the complex SL outperformed other machine learning algorithms. The complex SL had the highest predictive performance using predictors up to one-week post GDM diagnosis (discovery/validation set AUC: 0.934/0.815), followed by acceptably high predictability using predictors up to the time of GDM diagnosis (discovery/validation set AUC: 0.869/0.754), and relatively low predictability using predictors prior to GDM diagnosis (discovery/validation set AUC: 0.683–0.761/0.634–0.648). To improve interpretability and easiness of clinical uptake, we further devised a series of simpler logistic regression models using the top features selected in complex SL at each timing, which generated slightly lower but similar AUCs. Our population-based models could inform risk stratification of GDM treatment modality by assessing the risk of pharmacotherapy beyond MNT among pregnant individuals with GDM, with an ultimate goal to improve GDM care and management.

### Comparison with findings from previous studies

Despite studies that identified independent risk factors for GDM treatment (mostly insulin versus MNT alone) [21–27], studies focusing on the development of risk prediction models are limited, which further suffered from methodological limitations and collectively limited the generalizability of developed models. The low sensitivity (36%) of the model developed by Souza et al. may fail to adequately identify the group of patients requiring medication [28]. Barnes et al. developed a prediction model that yielded sensitivity of 86–93% for insulin therapy with the presence of 6–7 predictors [29]; however, given the wide span of the study period (1992–2015), the impact of different diagnostic criteria applied to future clinical practice is unknown [29]. Among other studies, model validation was lacking [28–32]. Further, for the two models which failed to predict insulin versus MNT alone [29, 30], the predictive ability may have been confined due to limited clinical variables (mostly glucose levels at diagnosis) and missing data on other potentially important socio-demographic, behavioral, and medical factors [22–24, 27].

In our study, the prediction methods employed were not able to achieve high discriminative performance using variables prior to pregnancy and before GDM diagnosis, suggesting the difficulty of assessing the risk of receiving pharmacologic treatment beyond MNT prior to diagnosis. Although identified as significant risk factors for GDM by previous studies [24, 28, 29] and our study, high pre-pregnancy BMI, prediabetes, and history of GDM did not present high predictive AUC results (best AUC performance: 0.634). Similarly, Pertot and colleagues reported the lack of predictive power for insulin therapy for GDM using maternal characteristics including race/ethnicity, BMI, family history of diabetes, hemoglobin A1c, and glucose levels [31]. Instead, the addition of week of gestational age at the diagnosis of GDM and glucose values at the diagnostic OGTT defined by the Carpenter-Coustan thresholds provide acceptably high AUC (0.752) using the calibrated simpler logistic regression model. Glycemic control measures during 1 week after diagnosis further increased the predictability of optimal treatment with a higher AUC (0.802); both prediction estimates were higher than those in previous studies with AUC around 0.700 [29, 30].

Physicians could use prediction models developed from GDM diagnosis and 1 week after GDM diagnosis to inform the patient-physician shared decisions regarding GDM treatment modality by balancing the 1-week difference in the timing of prediction (i.e., at the time of GDM diagnosis versus 1-week post diagnosis) against the slightly different predictive performance (difference in AUC: 0.05). The prediction models generated at the time of GDM diagnosis and 1 week after could be used to evaluate the likelihood of an eventual need of pharmacologic treatment. These tools could help facilitate the physician–patient conversation about the potential benefit, risk, and concerns of initiating pharmacologic treatment. The risk prediction models can play an essential role to improve efficiency of GDM care and management through the physician–patient joint discussion and decision making regarding GDM treatment modality. For patients requiring eventual pharmacologic treatment, reducing the waiting time between the first-line MNT and the optimal or last-line pharmacologic treatment (mean 5.6 weeks as observed herein within a window of 12–16 weeks for potential treatment) may result in a more effective intervention.

### Strengths and limitations

Our study has several notable strengths. To our best knowledge, this is the largest population-based study to date which developed and validated clinically oriented risk prediction models for GDM treatment modalities in a multi-racial/ethnic population. This is a sizeable increase compared to previous studies, with sample sizes ranging from 294 to 3317 [28–32]. We developed predictive models using supervised machine learning algorithms based on real-world data available in an integrated clinical setting at KPNC. The predictive models could be programmed into electronic health records of a health care delivery system to allow for automated risk stratification. Universal screening and diagnostic criteria for GDM applied uniformly throughout the study period (2007–2017) minimized misclassification bias. Further, pregnant individuals with GDM received universal referral to the KPNC Regional Perinatal Center for the supplemental care program beyond their standard of care. Both procedures minimized clinical practice variations, which was a major methodological limitation in previous studies [23, 24, 29]. Importantly, we performed rigorous tenfold cross-validation in the discovery and temporal/future validation sets to minimize the impact of data overfitting and bias selection.

Some potential limitations of our study merit discussion. The oracle properties of SL may only apply to the best algorithm within its selected library. Though the complex SL had a wide variety of adaptive and smooth learners, it is possible that there could be algorithms that could perform better outside of the current selection. Further, practical barriers may complicate the implementation of our predictive models in horizontally integrated or non-integrated health care systems, where data on glycemic control via self-monitoring of blood glucose collected following GDM diagnosis may not be readily available. Nonetheless, our calibrated simpler model using predictors at levels 1–3 (from 1-year prior to pregnancy to the time of GDM diagnosis; validation set AUC: 0.752) provided a slightly lower but still acceptably high AUC compared to prediction at levels 1–4 (up to 1 week post GDM diagnosis: validation set AUC: 0.802). Finally, our findings need further validation in populations from other health care delivery systems.

## Conclusions

The population-based, clinically oriented predictive models developed in this study for GDM treatment modality may provide the necessary support for the growing population of pregnant individuals with GDM to receive effective disease management in a timely fashion. Considering the clinical variables available at different stages of pregnancy, clinicians could assess the risk of receiving the more intensive pharmacotherapy beyond MNT at each timepoint. The series of simpler models developed based on the most influential features identified in the complex SL could be clinically friendly for uptake, with slightly lower but reasonably high predictive ability, compared to the complex SL with a full set of predictors. Timely conversation between health care providers and patients could be initiated to increase patient awareness of their likelihood of receiving pharmacotherapy beyond MNT. While the challenge lies in the length of time needed to monitor patients before prescribing antidiabetic oral agents or insulin, prediction models could potentially facilitate early triage to be incorporated into risk-based model of care and catalyze timelier and more effective GDM management. Before introducing these tools into a clinical care pathway, future work will need to focus on the development of clinical protocols suitable for use to conduct interventions and assess whether using these models result in patient benefits.

## Supplementary Information

**Additional file 1:** **Fig. S1.** Flow chart of the sequential treatment regime for gestational diabetes.

**Additional file 2:** **Table S1.** Detailed list of potential predictors extracted from electronic health records.

**Additional file 3:** **Table S2.** Out of bag imputation error estimates.

**Additional file 4:** **Table S3.** Treatment modality for gestational diabetes by calendar year.

**Additional file 5:** **Table S4.** 10-fold cross-validated AUC comparisons via Delong's test on the validation set.

**Additional file 6:** **Table S5.** Complex super learner output.

**Additional file 7:** **Table S6.** Simple super learner output.

**Additional file 8:** **Fig. S2.** Variable importance plots for CART and simple super learner algorithm using predictors available at A) level 1, B) levels 1-2, C) levels 1-3, and D) levels 1-4.

**Additional file 9:** **Table S7.** Interaction term(s) selection for the simplified logistic regression models built upon the most influential predictors from levels 1-4 selected from the complex super learner.

## Data Availability

Extracted data are available within the publication and its appendix. A de-identified analytic dataset used in this study can be shared with qualified researchers subject to approval by the Kaiser Foundation Research Institute Human Subjects Committee and by the Human Subjects Committee at the institutions requesting the data and a signed data sharing agreement. Please send all requests to the corresponding author of this article.

## Abbreviations

AUC: Area under the receiver operating characteristic curve
BMI: Body mass index
CART: Classification and regression tree
CI: Confidence interval
GCT: Glucose challenge test
GDM: Gestational diabetes mellitus
KPNC: Kaiser Permanente Northern California
LASSO: Least absolute shrinkage and selection operator
LMP: Last menstrual period
MNT: Medical nutrition therapy
OGTT: Oral glucose tolerance test
XGBoost: Extreme gradient boosting
