# Supplementary figures and images for: Development and validation of prediction models for gestational diabetes treatment modality using supervised machine learning: a population-based cohort study
Source: BMC Med. 2022 Sep 15;20:307. doi: 10.1186/s12916-022-02499-7 (PMC9476287; doi:10.1186/s12916-022-02499-7)

**Additional Figure 1. Flow chart of the sequential treatment regime for gestational diabetes**

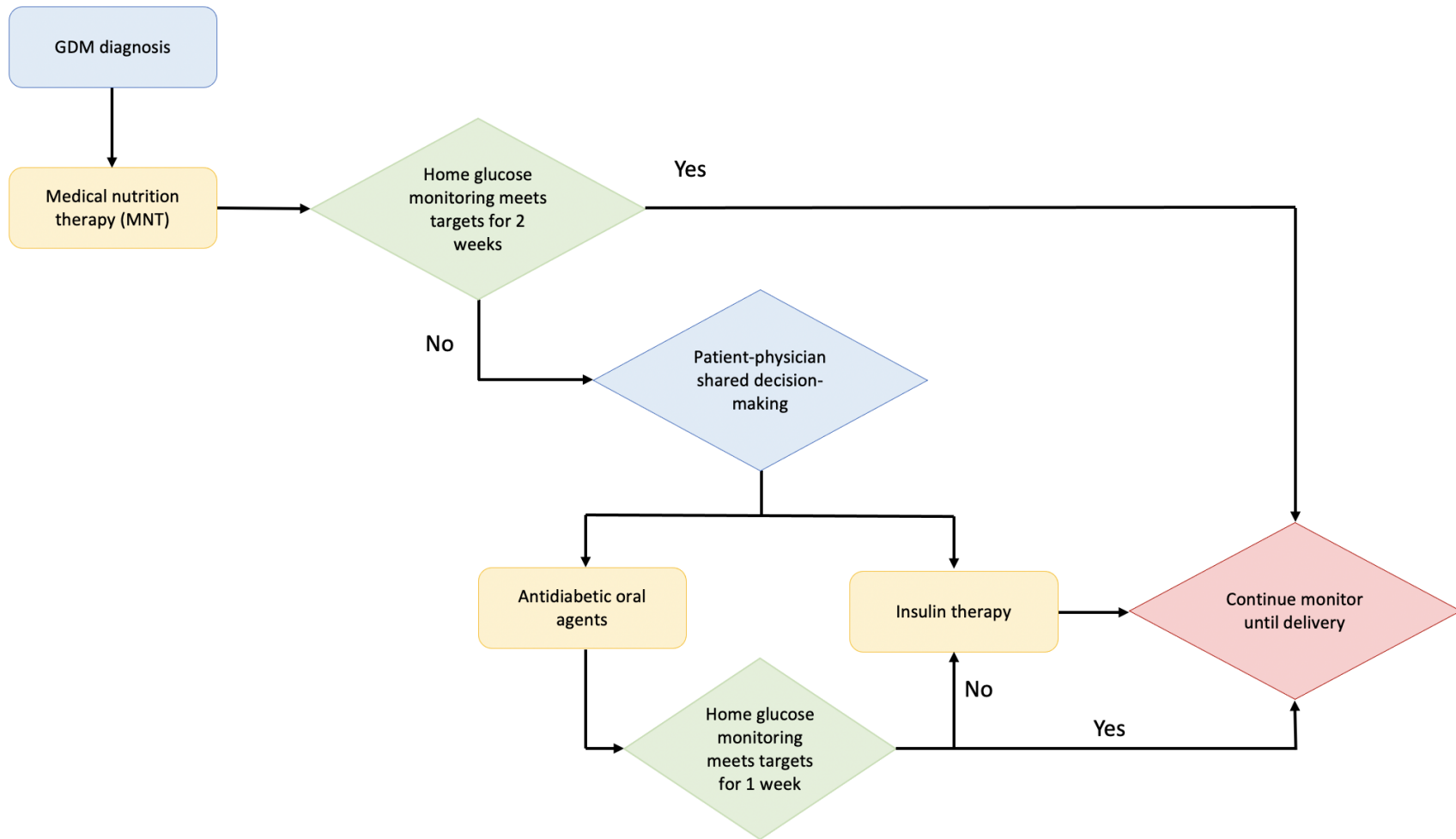

Supplement: Supplementary file 1 — Additional file 1: Fig. S1. Flow chart of the sequential treatment regime for gestational diabetes. [file 12916_2022_2499_MOESM1_ESM.pdf]
