# Supplementary material for: Development and validation of prediction models for gestational diabetes treatment modality using supervised machine learning: a population-based cohort study
Source: BMC Med. 2022 Sep 15;20:307. doi: 10.1186/s12916-022-02499-7 (PMC9476287; doi:10.1186/s12916-022-02499-7)
Supplement: Supplementary file 2 — Additional file 2: Table S1. Detailed list of potential predictors extracted from electronic health records. [file 12916_2022_2499_MOESM2_ESM.pdf]

Additional Table 1: Detailed list of potential predictors extracted from electronic health records

Timing	Type	Predictors	Missing Rate, %
1. One year prior to the index pregnancy to LMP	Birth information	Year pregnancy initiated	0.0%
		Birth counts recorded as singleton, twin, or multiples	0.0%
	Demographics	Age of mother at delivery	0.0%
		Race/ethnicity	0.0%
		Race/ethnicity defined by Kaiser Permanente Northern California categories	0.0%
		Hispanic or Latino ethnicity	0.0%
		Highest education obtained by the mother	8.2%
		≥6 months gap in Kaiser Permanente membership	0.0%
		Median family income numeric	0.7%
		Median family income categorical	0.7%
		Median household income numeric	0.7%
		Median household income categorical	0.7%
		Parity excluding current pregnancy	0.0%
	Lifestyle/behavioral factors 12 months before pregnancy	Nicotine (i.e. cigarettes, nicorette gum) recorded as daily, weekly, or never	0.0%
		Nicotine (i.e. cigarettes, nicorette gum) recorded as ever or never	0.0%
		Alcohol (i.e. wine, beer, liquor) recorded as daily, weekly, or never	0.0%
		Alcohol (i.e. wine, beer, liquor) recorded as ever or never	0.0%
		Exercise days per week prior to conception	62.8%
		Exercise days per week prior to conception in three categories	62.8%
		Exercise minutes per day prior to conception	62.9%
		Exercise minutes per days prior to conception in four categories	62.9%
		Exercise minutes per week prior to conception	62.9%
		Exercise minutes per week prior to conception in three categories	62.9%
		Anemia by diagnosis	0.0%

	Use of antihyperlipidemic medication	0.0%
	Use of antihypertensive medications[1]	0.0%
	Arrythmia by diagnosis	0.0%
	Asthma by diagnosis	0.0%
	Malignant cancer prior to GDM	0.0%
	Benign/borderline/in situ cancer prior to GDM	0.0%
	Cerebrovascular disease by diagnosis	0.0%
	Cerebrovascular disease by procedure	0.0%
	Cerebrovascular disease by diagnosis or procedure	0.0%
	Coronary heart disease by diagnosis	0.0%
	Coronary heart disease by procedure	0.0%
	Coronary heart disease by diagnosis or procedure	0.0%
	Chronic heart failure by diagnosis	0.0%
	Pre-existing depression	0.0%
	Dyslipidemia by diagnosis	0.0%
	Dyslipidemia by diagnosis or medications	0.0%
Pre-existing conditions/family history	Family history of diabetes by diagnosis	0.0%
	Hypertension diagnosed[1]	0.0%
	History of abortive outcome (ICD9:630-639, ICD10:O00-O08)	0.0%
	Previous macrosomia (>4.5kg)	0.0%
	History of gestational diabetes	0.0%
	Chronic kidney disease by diagnosis	0.0%
	Polycystic ovary syndrome by diagnosis	0.0%
	Peripheral vascular diseases by diagnosis	0.0%
	Peripheral vascular diseases by procedure	0.0%
	Peripheral vascular diseases by diagnosis or procedure	0.0%
	Fasting plasma glucose value 100-125 mg/dL	0.0%
	2-hour post challenge plasma glucose value 140-199 mg/dL	0.0%
	HbA1C value 5.7-6.5%	0.0%
	Chronic obstructive pulmonary disease by diagnosis	0.0%
	Pre-existing prediabetes[1] diagnosis by lab and ICD codes	0.0%
Typical measures	Height	0.2%

		Weight	7.2%
		Pre-pregnancy body mass index	7.3%
		Highest triglyceride preconception ≥200 mg/dl	0.0%
		Highest triglyceride during pregnancy ≥200 mg/dl	0.0%
		Highest Cholesterol preconception ≥240 mg/dl	0.0%
		Highest Cholesterol pregnancy ≥240 mg/dl	0.0%
		Lowest HDL preconception <40 mg/dl	0.0%
		Lowest HDL during pregnancy <40 mg/dl	0.0%
		Highest LDL preconception ≥160 mg/dl	0.0%
		Highest LDL during pregnancy ≥160 mg/dl	0.0%
		Average diastolic blood pressure[1]	28.4%
		Average systolic blood pressure[1]	28.4%
2. LMP to before GDM diagnosis	Baby information	Sex of the baby	1.2%
	Lifestyle/Habits during pregnancy	Nicotine (i.e. cigarettes, Nicorette gum) recorded as daily, weekly, or never	0.0%
		Nicotine (i.e. cigarettes, Nicorette gum) recorded as ever or never	0.0%
		Alcohol (i.e. wine, beer, liquor) recorded as daily, weekly, or never	0.0%
		Alcohol (i.e. wine, beer, liquor) recorded as ever or never	0.0%
		Exercise days per week during pregnancy prior to GDM	46.0%
		Exercise days per week during pregnancy prior to GDM in three categories	46.0%
		Exercise minutes per day during pregnancy prior to GDM	46.1%
		Exercise minutes per days during pregnancy prior to GDM in four categories	46.1%
		Exercise minutes per week during pregnancy prior to GDM	0.0%
	Typical clinical measures/medications	Last pregnancy weight prior to GDM	3.5%
		Difference in days between last weight measured and GDM diagnosis	3.5%
		Depression diagnosis during pregnancy	0.0%
		Use of antihypertensive medications	0.0%

		Average diastolic blood pressure in pregnancy	0.0%
		Average systolic blood pressure in pregnancy	0.0%
		Max diastolic blood pressure in pregnancy	0.0%
		Max systolic blood pressure in pregnancy	0.0%
		Median diastolic blood pressure in pregnancy	0.0%
		Median systolic blood pressure in pregnancy	0.0%
		Count of blood pressure in pregnancy	0.0%
		Systolic blood pressure close to GDM diagnosis	3.0%
		Diastolic blood pressure close to GDM diagnosis	3.0%
		Gestational weight gain up to GDM date	7.7%
		Screening value of glucose test not used for diagnosis	3.3%
		Hypertension diagnosis	0.0%
3. At GDM diagnosis	GDM diagnostic test	Week of GDM Diagnosis	0.0%
		Fasting glucose value from the 3-hr OGTT	10.9%
		Fasting glucose value from the 3-hr OGTT, <95 mg/dL	10.9%
		One-hour glucose value from the 3-hr OGTT	10.8%
		One-hour glucose value from the 3-hr OGTT, <180 mg/dL	10.8%
		Two-hour glucose value from the 3-hr OGTT	10.8%
		Two-hour glucose value from the 3-hr OGTT, <155 mg/dL	10.8%
		Three-hour glucose value from the 3-hr OGTT	10.7%
		Three-hour glucose value from the 3-hr OGTT, <140 mg/dL	10.7%
		Abnormal value by the Carpenter and Coustan criteria[2]	0.0%
		Gestational weeks at GDM diagnosis (continuous)	0.0%
		Measure of severity gathered from the 3-hr OGTT with at least 2 values from the fasting, 1-hour, 2-hour, 3-hour glucose	0.0%

		levels above 1 standard deviation of the respective mean	
4. One week following GDM diagnosis	Glycemic control monitoring	Mean glucose test 1-hour after meal (breakfast, lunch, or dinner)	7.9%
		Mean glucose test 1-hour after breakfast	8.2%
		Mean glucose test 1-hour after lunch	8.3%
		Mean glucose test 1-hour after dinner	8.4%
		Mean fasting glucose	7.9%
		Met the goal of ≥80% of glucose tests below 140 mg/dL 1-hour after meal	7.9%
		Met the goal of ≥80% of glucose tests below 140 mg/dL 1-hour after breakfast	0.0%
		Met the goal of ≥80% of glucose tests below 140 mg/dL 1-hour after lunch	0.0%
		Met the goal of ≥80% of glucose tests below 140 mg/dL 1-hour after dinner	0.0%
		≥80% fasting glucose below or at 95 and ≥80% of 1-hour postprandial glucose test below or at 140 mg/dL	0.0%
		Met the goal of ≥80% of all fasting and 1-hour postprandial glucose tests below cutoff point	0.0%
		Met the goal of ≥80% of glucose tests below 95 mg/dL	0.0%
		Count of glucose test 1-hr after meal (breakfast, lunch, or dinner)	7.9%
		Count of glucose test 1-hr after breakfast	8.2%
		Count of glucose test 1-hr after lunch	8.3%
		Count of glucose test 1-hr after dinner	8.4%
		Count of glucose test 1-hour after meal <140 mg/dL	7.9%
		Count of glucose test 1-hour after breakfast <140 mg/dL	8.2%
		Count of glucose test 1-hour after lunch <140 mg/dL	8.3%
		Count of glucose test 1-hour after dinner <140 mg/dL	8.4%
		Count of fasting glucose <95 mg/dL	7.9%
		Count of fasting glucose	7.9%
		% all fasting and 1-hr postprandial glucose less than the cutoff point	7.8%

	% all fasting and 1-hr/2-hr postprandial glucose less than the cutoff point	7.8%
	% glucose test 1-hour after meal <140 mg/dL	7.9%
	% glucose test 1-hour after breakfast <140 mg/dL	8.2%
	% glucose test 1-hour after lunch <140 mg/dL	8.4%
	% glucose test 1-hour after dinner <140 mg/dL	8.3%
	% fasting glucose <95 mg/dL	7.9%
	Met the goal of ≥80% of glucose tests below 140 mg/dL 1-hour	7.9%
	Met the goal of ≥80% of glucose tests below 140 mg/dL 1-hour breakfast	0.0%
	Met the goal of ≥80% of glucose tests below 140 mg/dL 1-hour lunch	0.0%
	Met the goal of ≥80% of glucose tests below 140 mg/dL 1-hour dinner	0.0%
	Met 1-hour post lunch glycemic control ≥80% during 1st week of initiation	15.6%
	Met 1-hour post dinner glycemic control ≥80% during 1st week of initiation	15.6%
	Met 1-hour post breakfast glycemic control ≥80% during 1st week of initiation	15.6%
	Met glycemic control ≥80% during 1st week of initiation	15.6%
	Met fasting glycemic control ≥80% during first week of initiation	15.6%
	Mean glucose test 1-hour after breakfast during 1st week of initiation	17.0%
	Mean glucose test 1-hour after lunch during 1st week of initiation	17.1%
	Mean glucose test 1-hour after dinner during 1st week of initiation	17.2%
	Mean fasting glucose during 1st week of initiation	16.4%
	Count of 1-hour post lunch glycemic control measured during 1st week of initiation	17.1%
	Count of 1-hour post dinner glycemic control measured during 1st week of initiation	17.2%

	Count of 1-hour post breakfast glycemic control measured during 1st week of initiation	17.0%
	Count of fasting glucose test measured during 1st week of initiation	16.4%
	Count of fasting, 1hr/2hr postprandial glucose tests measured during 1st week of initiation	15.6%
	Count of tests that met the goal for 1-hour post lunch glycemic control measured during 1st week of initiation	17.2%
	Count of tests that met the goal for 1-hour post dinner glycemic control measured during 1st week of initiation	17.1%
	Count of tests that met the goal for 1-hour post breakfast glycemic control measured during 1st week of initiation	17.0%
	Count of tests that met the goal for fasting glucose test measured during 1st week of initiation	16.4%
	Count of tests that met the goal for fasting, 1hr/2hr postprandial glucose tests measured during 1st week of initiation	15.6%
	Count of tests that met the goal for fasting glucose test measured during 1st week of initiation	16.4%
	Count of tests that met the goal for fasting, 1hr/2hr postprandial glucose tests measured during 1st week of initiation	15.6%
	Count of tests that met the goal for fasting glucose test measured during 1st week of initiation	16.4%
	Count of tests that met the goal for fasting, 1hr/2hr postprandial glucose tests measured during 1st week of initiation	15.6%
	Count of tests that met the goal for fasting glucose test measured during 1st week of initiation	16.4%
	Count of tests that met the goal for fasting, 1hr/2hr postprandial glucose tests measured during 1st week of initiation	15.6%

	Count of tests that met the goal for fasting glucose test measured during 1st week of initiation	16.4%
	Count of tests that met the goal for fasting, 1hr/2hr postprandial glucose tests measured during 1st week of initiation	15.6%
