# Supplementary material for: Development and validation of prediction models for gestational diabetes treatment modality using supervised machine learning: a population-based cohort study
Source: BMC Med. 2022 Sep 15;20:307. doi: 10.1186/s12916-022-02499-7 (PMC9476287; doi:10.1186/s12916-022-02499-7)
Supplement: Supplementary file 3 — Additional file 3: Table S2. Out of bag imputation error estimates. [file 12916_2022_2499_MOESM3_ESM.pdf]

Additional Table 2. Out of bag imputation error estimates

Year	NRMSE	PFC
2007	0.0012	0.0239
2008	0	0.0249
2009	0.0013	0.0254
2010	0.0013	0.0255
2011	0.0013	0.0247
2012	0.0013	0.0255
2013	0	0.0259
2014	0.0013	0.0256
2015	0.0013	0.0267
2016	0.0013	0.0253
2017	0.0013	0.0256

NRMSE, normalized root mean squared error; PFC, proportion of falsely classified entries
