# Supplementary material for: Development and validation of prediction models for gestational diabetes treatment modality using supervised machine learning: a population-based cohort study
Source: BMC Med. 2022 Sep 15;20:307. doi: 10.1186/s12916-022-02499-7 (PMC9476287; doi:10.1186/s12916-022-02499-7)
Supplement: Supplementary file 4 — Additional file 4: Table S3. Treatment modality for gestational diabetes by calendar year. [file 12916_2022_2499_MOESM4_ESM.pdf]

Additional Table 3: Treatment modality for gestational diabetes by calendar year

	All *n* = 30,474	2007 n = 2598	2008 n = 2566	2009 n = 2846	2010 n = 2571	2011 n = 2491	2012 n = 2616	2013 n = 2548	2014 n = 2911	2015 n = 3152	2016 n = 2941	2017 n = 3234	*P* value[1]
MNT	18657 (61.2)	1968 (75.8)	1941 (75.6)	2008 (70.6)	1609 (62.6)	1695 (68.0)	1740 (66.5)	1387 (54.4)	1832 (62.9)	1582 (50.2)	1189 (40.4)	1706 (52.8)	0.9995
Antidiabetic oral agents	9942 (32.6)	469 (18.1)	500 (19.5)	705 (24.8)	819 (31.9)	645 (25.9)	739 (28.2)	985 (38.7)	938 (32.2)	1394 (44.2)	1469 (49.9)	1279 (39.5)	0.0003
Insulin therapy	1875 (6.2)	161 (6.2)	125 (4.9)	133 (4.7)	143 (5.6)	151 (6.1)	137 (5.2)	176 (6.9)	141 (4.8)	176 (5.6)	283 (9.6)	249 (7.7)	0.0801

[1]Obtained by Mann-Kendall test; the alternative hypothesis states there exists a monotonically increasing trend.
