# Supplementary material for: Development and validation of prediction models for gestational diabetes treatment modality using supervised machine learning: a population-based cohort study
Source: BMC Med. 2022 Sep 15;20:307. doi: 10.1186/s12916-022-02499-7 (PMC9476287; doi:10.1186/s12916-022-02499-7)
Supplement: Supplementary file 5 — Additional file 5: Table S4. 10-fold cross-validated AUC comparisons via Delong's test on the validation set. [file 12916_2022_2499_MOESM5_ESM.pdf]

Additional Table 4: 10-fold cross-validated AUC comparisons via Delong's test on the validation set

Prediction Method	Predictor Levels[1]	P-values
CART	1-2 vs. 1	0.0127
	1-3 vs. 1-2	<2.2e-16
	1-4 vs. 1-3	8.495E-08
LASSO regression	1-2 vs. 1	0.0041
	1-3 vs. 1-2	<2.2e-16
	1-4 vs. 1-3	<2.2e-16
Simple super learner[2]	1-2 vs. 1	0.0103
	1-3 vs. 1-2	<2.2e-16
	1-4 vs. 1-3	<2.2e-16
Complex super learner[3]	1-2 vs. 1	0.0008
	1-3 vs. 1-2	<2.2e-16
	1-4 vs. 1-3	<2.2e-16

AUC, area under the curve; CART, classification and regression tree; LASSO, least absolute shrinkage and selection operator; XGBoost, extreme gradient boosting

[1]Level 1: one-year preconception to last menstrual period; level 2: last menstrual period to before diagnosis of gestational diabetes; level 3: at the time of diagnosis of gestational diabetes; level 4: one week following diagnosis of gestational diabetes.

[2]Candidate algorithms in simple super learner included response-mean, LASSO regression, and CART.

[3]Candidate algorithms in complex super learner included response-mean, LASSO regression, CART, random forest, and XGBoost algorithms.
