# Supplementary material for: Development and validation of prediction models for gestational diabetes treatment modality using supervised machine learning: a population-based cohort study
Source: BMC Med. 2022 Sep 15;20:307. doi: 10.1186/s12916-022-02499-7 (PMC9476287; doi:10.1186/s12916-022-02499-7)
Supplement: Supplementary file 6 — Additional file 6: Table S5. Complex super learner output. [file 12916_2022_2499_MOESM6_ESM.pdf]

Additional Table 5: Complex super learner output

**Predictors level**	**Candidate algorithms**	**Risk[1]**	**Coefficient**
	Response-mean	0.501	0.027
	LASSO regression	0.331	0.301
Level 1	CART	0.393	0
	Random forest	0.351	0
	XGBoost	N/A[2]	0.673
	Response-mean	0.501	0.033
	LASSO regression	0.316	0.379
Levels 1-2	CART	0.387	0
	Random forest	0.330	0.050
	XGBoost	N/A[2]	0.539
	Response-mean	0.501	0.027
	LASSO regression	0.214	0.223
Levels 1-3	CART	0.264	0
	Random forest	0.215	0.080
	XGBoost	N/A[2]	0.670
	Response-mean	0.501	0.035
	LASSO regression	0.151	0.190
Levels 1-4	CART	0.219	0
	Random forest	0.146	0.164
	XGBoost	N/A[2]	0.611

CART, classification and regression trees; LASSO, least absolute shrinkage and selection operator; XGBoost, extreme gradient boosting

[1]Risk is the mean of the loss measured by the negative binomial log-likelihood

[2]N/A for XGBoost due to multiple tuning parameters used in the candidate learners set. Risk over multiple XGBoost algorithms is not shown.
