# Supplementary material for: Development and validation of prediction models for gestational diabetes treatment modality using supervised machine learning: a population-based cohort study
Source: BMC Med. 2022 Sep 15;20:307. doi: 10.1186/s12916-022-02499-7 (PMC9476287; doi:10.1186/s12916-022-02499-7)
Supplement: Supplementary file 7 — Additional file 7: Table S6. Simple super learner output. [file 12916_2022_2499_MOESM7_ESM.pdf]

Additional Table 6: Simple super learner output

Candidate algorithms	Risk[1]	Coefficient
Response-mean	0.501	0.372
LASSO regression	0.331	0.628
CART	0.393	0
Response-mean	0.501	0.344
LASSO regression	0.316	0.648
CART	0.387	0.008
Response-mean	0.501	0.375
LASSO regression	0.214	0.537
CART	0.264	0.088
Response-mean	0.501	0.334
LASSO regression	0.151	0.588
CART	0.219	0.077

CART, classification and regression trees; LASSO, least absolute shrinkage and selection operator.

[1]Risk is the mean of the loss measured by the negative binomial log-likelihood
