# Supplementary material for: Development and validation of prediction models for gestational diabetes treatment modality using supervised machine learning: a population-based cohort study
Source: BMC Med. 2022 Sep 15;20:307. doi: 10.1186/s12916-022-02499-7 (PMC9476287; doi:10.1186/s12916-022-02499-7)
Supplement: Supplementary file 8 — Additional file 8: Fig. S2. Variable importance plots for CART and simple super learner algorithm using predictors available at A) level 1, B) levels 1-2, C) levels 1-3, and D) levels 1-4. [file 12916_2022_2499_MOESM8_ESM.pdf]

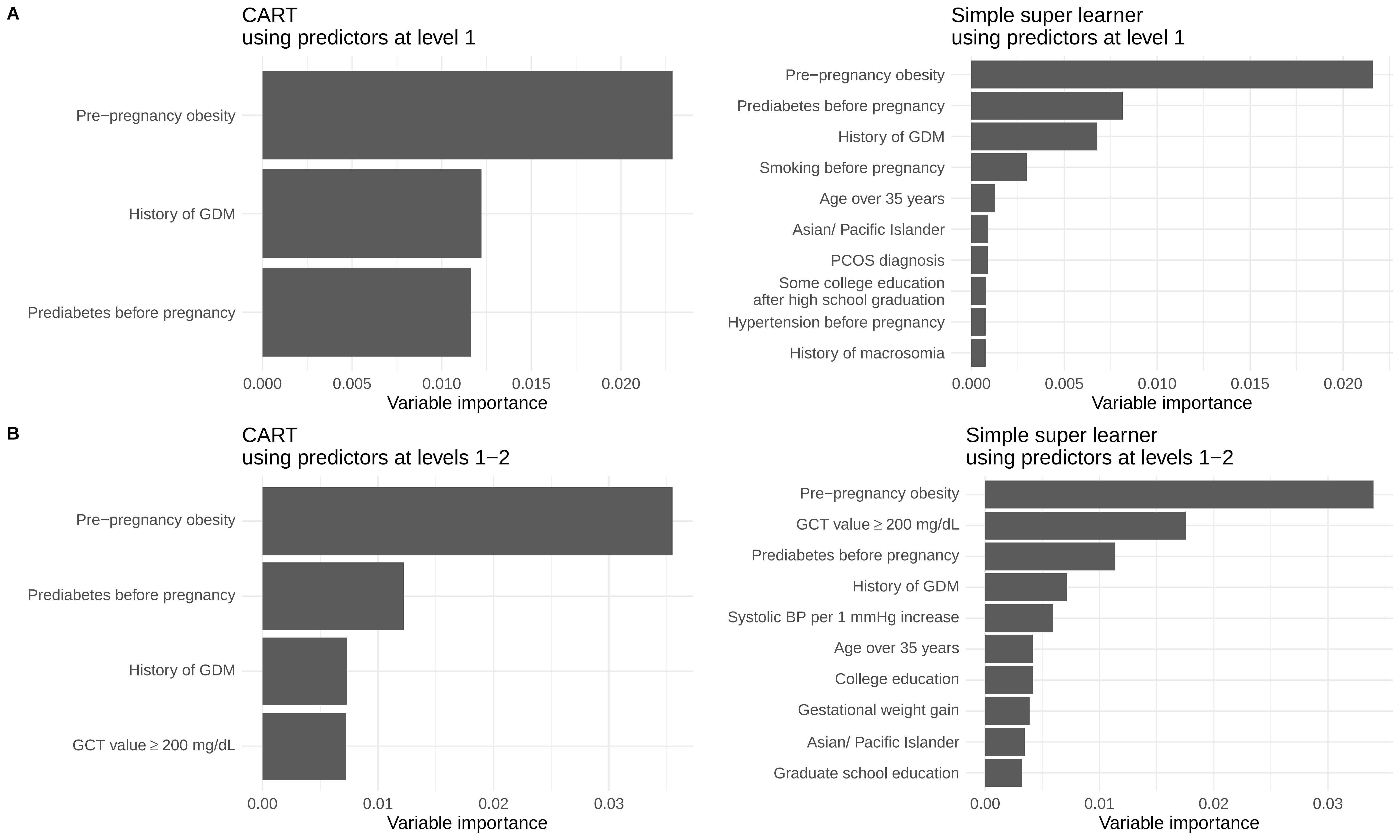

A
CART
using predictors at level 1
Pre−pregnancy obesity
History of GDM
Prediabetes before pregnancy
0.000
0.005
0.010
0.015
0.020
Variable importance
Simple super learner
using predictors at level 1
Pre−pregnancy obesity
Prediabetes before pregnancy
History of GDM
Smoking before pregnancy
Age over 35 years
Asian/ Pacific Islander
PCOS diagnosis
Some college education
after high school graduation
Hypertension before pregnancy
History of macrosomia
0.000
0.005
0.010
0.015
0.020
Variable importance
B
CART
using predictors at levels 1−2
Pre−pregnancy obesity
Prediabetes before pregnancy
History of GDM
GCT value ≥ 200 mg/dL
0.00
0.01
0.02
0.03
Variable importance
Simple super learner
using predictors at levels 1−2
Pre−pregnancy obesity
GCT value ≥ 200 mg/dL
Prediabetes before pregnancy
History of GDM
Systolic BP per 1 mmHg increase
Age over 35 years
College education
Gestational weight gain
Asian/ Pacific Islander
Graduate school education
0.00
0.01
0.02
0.03
Variable importance

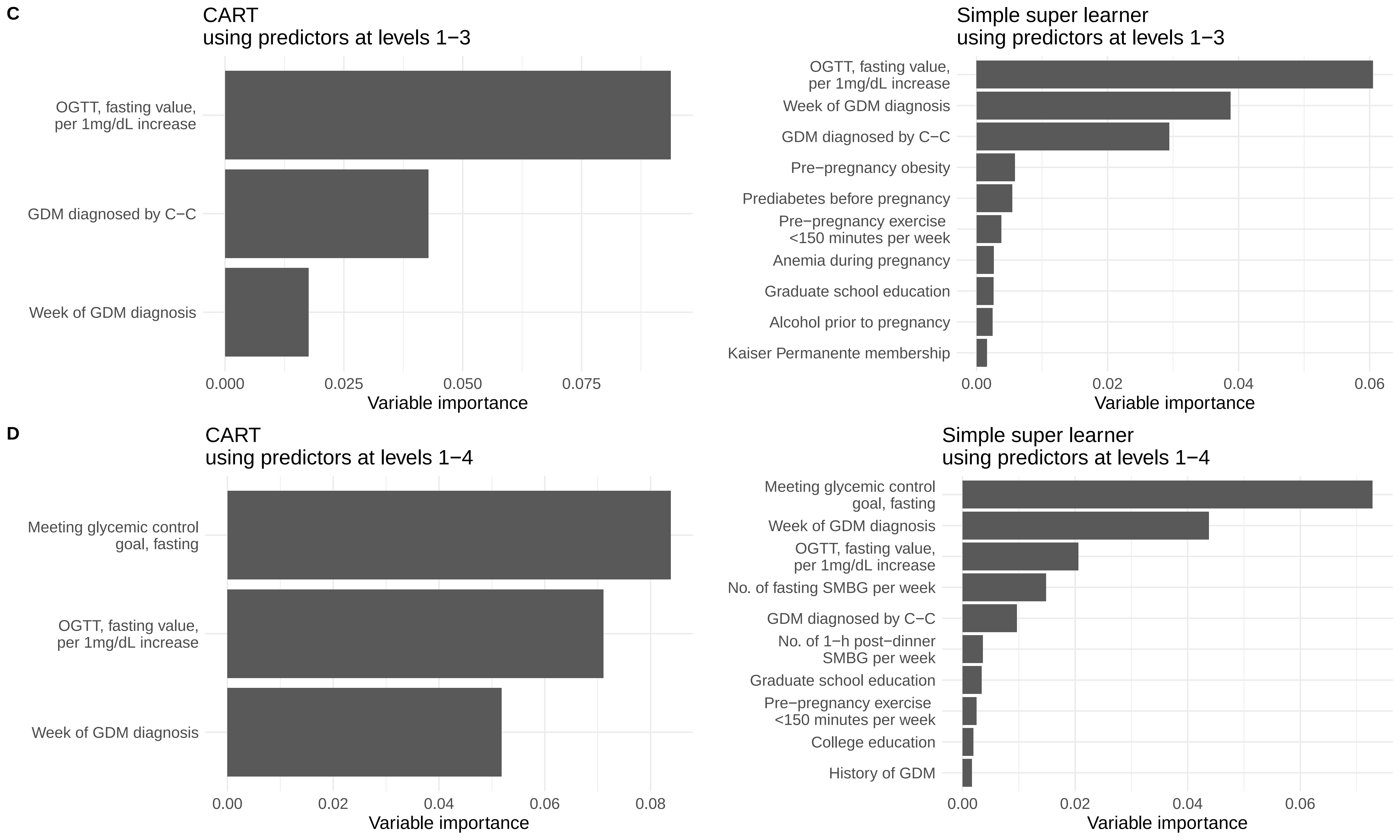
C
CART
using predictors at levels 1−3
OGTT, fasting value, per 1mg/dL increase
GDM diagnosed by C−C
Week of GDM diagnosis
0.000
0.025
0.050
0.075
Variable importance
Simple super learner
using predictors at levels 1−3
OGTT, fasting value, per 1mg/dL increase
Week of GDM diagnosis
GDM diagnosed by C−C
Pre−pregnancy obesity
Prediabetes before pregnancy
Pre−pregnancy exercise <150 minutes per week
Anemia during pregnancy
Graduate school education
Alcohol prior to pregnancy
Kaiser Permanente membership
0.00
0.02
0.04
0.06
Variable importance
D
CART
using predictors at levels 1−4
Meeting glycemic control goal, fasting
OGTT, fasting value, per 1mg/dL increase
Week of GDM diagnosis
0.00
0.02
0.04
0.06
0.08
Variable importance
Simple super learner
using predictors at levels 1−4
Meeting glycemic control goal, fasting
Week of GDM diagnosis
OGTT, fasting value, per 1mg/dL increase
No. of fasting SMBG per week
GDM diagnosed by C−C
No. of 1−h post−dinner SMBG per week
Graduate school education
Pre−pregnancy exercise <150 minutes per week
College education
History of GDM
0.00
0.02
0.04
0.06
Variable importance
