# Supplementary material for: Development and validation of prediction models for gestational diabetes treatment modality using supervised machine learning: a population-based cohort study
Source: BMC Med. 2022 Sep 15;20:307. doi: 10.1186/s12916-022-02499-7 (PMC9476287; doi:10.1186/s12916-022-02499-7)
Supplement: Supplementary file 9 — Additional file 9: Table S7. Interaction term(s) selection for the simplified logistic regression models built upon the most influential predictors from levels 1-4 selected from the complex super learner. [file 12916_2022_2499_MOESM9_ESM.pdf]

Additional Table 7: Interaction term(s) selection for the simplified logistic regression models built upon the most influential predictors from levels 1-4 selected from the complex super learner

	Number of main terms and interaction terms included	10-fold CV-AUC in the validation set
Full discovery set	14	0.800 (0.785-0.815)
Model 1	12	0.800 (0.785-0.815)
Model 2	9	0.800 (0.785-0.815)
Model 3	9	0.801 (0.786-0.816)
Model 4	8	0.798 (0.783-0.813)
Model 5	14	0.799 (0.783-0.814)
Model 6	11	0.795 (0.780-0.811)
Model 7	11	0.803 (0.788-0.818)
Model 8	11	0.800 (0.785-0.816)
Model 9	16	0.798 (0.783-0.813)
Model 10	6	0.794 (0.779-0.810)

CV-AUC, cross validated area under the receiver operating characteristic curve.
The models 1-10 each represents stepwise regression fit on a tenth of the discovery set (non-overlapping).
